# P-1989. COVID-19 Mortality Among Immunocompromised Individuals is Consistently High Compared with Non-Immunocompromised Individuals: Results of the INFORM Study 2022, England

**DOI:** 10.1093/ofid/ofae631.2147

**Published:** 2025-01-29

**Authors:** Lucy Carty, Sabada Dube, Carla Talarico, Richard McNulty, Nahila Justo, Renata Yokota, Michelle Harley, Jurgens Peters, Kathryn Evans, Yi Lu, Sylvia Taylor, Jennifer Quint, Rachael A Evans

**Affiliations:** AstraZeneca, Cambridge, England, United Kingdom; Medical Evidence, Vaccines and Immune Therapies Unit, AstraZeneca, Cambridge, England, United Kingdom; Vaccines and Immune Therapies, BioPharmaceuticals Medical, AstraZeneca, Gaithersburg, MD, USA, Gaithersburg, Maryland; Medical Affairs, Vaccines and Immune Therapies Unit, AstraZeneca, Cambridge, UK, Cambridge, England, United Kingdom; Real-World Evidence, Data Analytics, Evidera, Stockholm, Sweden and Department of Neurobiology, Care Science and Society, Karolinska Institute, Stockholm, Sweden, Stockholm, Sodermanlands Lan, Sweden; P95, Leuven, Belgium, Dilbeek, Luxembourg, Belgium; AstraZeneca, Cambridge, England, United Kingdom; AstraZeneca, Cambridge, England, United Kingdom; Real-World Evidence, Data Analytics, Evidera, Waltham, MA, USA, Waltham, Massachusetts; Real-World Evidence, Data Analytics, Evidera, London, UK, London, England, United Kingdom; Medical Evidence, Vaccines and Immune Therapies Unit, AstraZeneca, Cambridge, UK, Cambridge, England, United Kingdom; National Heart and Lung Institute, Imperial College London, London, UK, Cambridge, England, United Kingdom; University of Leicester, Leicester, England, United Kingdom

## Abstract

**Background:**

The INFORM study showed the disproportionate risk of COVID-19 hospitalization and mortality among immunocompromised (IC) versus non-immunocompromised (non-IC) individuals. This analysis aimed to describe the relationship between COVID-19 hospitalization and COVID-19 mortality by IC status.

**Methods:**

The INFORM retrospective cohort study assessed electronic healthcare records from a random 25% sample of the population of England, aged ≥ 12 years, who were registered and active in the National Health Service. Individuals were categorized by COVID-19 hospitalization status and highest level of in-hospital care: no intensive care unit (ICU) admission or mechanical ventilation (MV); ICU without MV; and ICU with MV. COVID-19 deaths included those with COVID-19 recorded as the cause of death and/or in-hospital death during COVID-19 hospitalization (admission for > 24 hours with COVID-19 as the primary diagnosis) from January 1 to December 31, 2022.

**Results:**

In 2022, IC individuals comprised 3.9% of the study population (N = 470,910/11,990,730). Of those with a recorded SARS-CoV-2 infection (N=1,746,820), COVID-19 deaths occurred in 1.1% of IC and 0.2% of non-IC individuals. Of those with a recorded COVID-19 hospitalization (N=20,995), COVID-19 deaths occurred in 12.0% of IC and 9.3% of non-IC individuals.

There was a higher proportion of COVID-19 deaths, either during or after admission, among hospitalized IC individuals compared with non-IC individuals across all levels of care: no ICU and/or MV, 10.7% versus 8.6%; ICU, 48.1% versus 36.7%; MV, 77.8% versus 50.8%.

**Conclusion:**

Among those who required hospitalization for COVID-19, the proportion who died of COVID-19 during or after admission was consistently high among IC individuals compared with non-IC individuals across all levels of care, further highlighting the disproportionate burden of COVID-19 in this vulnerable population.

**Disclosures:**

Lucy Carty, PhD, AstraZeneca: Employee of, and may hold stock and/or stock options Sabada Dube, PhD, AstraZeneca: Employee of, and may hold stock and/or stock options Carla Talarico, PhD, MPH, AstraZeneca: Employee of AstraZeneca and may hold stock and/or stock options Richard McNulty, MD, AstraZeneca: Employee of, and may hold stock and/or stock options Nahila Justo, PhD, MBA, Evidera/AstraZeneca: Employee of Evidera, which received funding for the conduct of the current study from AstraZeneca Renata Yokota, PhD, P95 Epidemiology & Pharmacovigilance/AstraZeneca: Employee of P95 Epidemiology & Pharmacovigilance, which received a consultancy fee for the conduct of the current study from AstraZeneca Michelle Harley, MBBS, MRCP, MRCGP, AstraZeneca: Employee, holds or may hold stock Jurgens Peters, MD, MPH, MSc, MBA, AstraZeneca: Employee, holds or may hold stock Kathryn Evans, MPH, Evidera/AstraZeneca: Employee of Evidera, which received funding for the conduct of the current study from AstraZeneca Yi Lu, PhD, Evidera/AstraZeneca: Employee of Evidera, which received funding for the conduct of the current study from AstraZeneca Sylvia Taylor, PhD, MPH, MBA, AstraZeneca: Employee of, and may hold stock and/or stock options in, AstraZeneca Jennifer Quint, PhD, Asthma + Lung UK: Grant/Research Support|AstraZeneca: Advisor/Consultant|AstraZeneca: Grant/Research Support|Boehringer Ingelheim: Grant/Research Support|Evidera: Advisor/Consultant|Evidera: Funding|GSK: Advisor/Consultant|GSK: Grant/Research Support|Health Data Research UK (HDR UK): Grant/Research Support|Insmed: Advisor/Consultant|Medical Research Council (MRC): Grant/Research Support Rachael A. Evans, PhD FRCP, Boehringer Ingelheim: Speaker fees|Chiesi: Support for attending the British Thoracic Society (BTS)|Chiesi: Serving non-paid as the European Respiratory Society (ERS) Group 01.02 Pulmonary Rehabilitation and Chronic Care secretary|Chiesi: Serving non-paid as the American Thoracic Society (ATS) Pulmonary Rehabilitation Assembly chairperson|Evidera: Funding for providing advice throughout the current study|Genetec/Roche: Grant/Research Support|Moderna: Speaker fees|NIHR: Grant/Research Support|UKRI: Grant/Research Support|Wolfson Foundation: Grant/Research Support

